# The Dysregulation of the Renin–Angiotensin System in COVID-19 Studied by Serum Proteomics: Angiotensinogen Increases with Disease Severity

**DOI:** 10.3390/molecules27082495

**Published:** 2022-04-12

**Authors:** Phil-Robin Tepasse, Richard Vollenberg, Nico Steinebrey, Simone König

**Affiliations:** 1 Department of Medicine B for Gastroenterology, Hepatology, Endocrinology and Clinical Infectiology, University Hospital Münster, 48149 Münster, Germany; phil-robin.tepasse@ukmuenster.de (P.-R.T.); richard.vollenberg@ukmuenster.de (R.V.); 2 IZKF Core Unit Proteomics, University of Münster, 48149 Münster, Germany; n.steinebrey@uni-muenster.de

**Keywords:** ACE, CPN, KLKB1, KNG1, AGT, bradykinin, coronavirus, RAS, proteomics, mass spectrometry, expression analysis, serum

## Abstract

(1) Background: ACE and CPN serum activity correlated with disease severity in an earlier study of 45 hospitalized COVID-19 patients. The serum protein profile was investigated in the same cohort here to shed more light on the involvement of the renin–angiotensin system (RAS). (2) Methods: High-definition mass spectrometry-based protein expression analysis was performed, followed by multivariate statistical and network analyses. (3) Results: The protein profiles of hospitalized patients (HoP) differed significantly from those of convalescent and healthy probands. Surprisingly, HoP samples separated into six groups according to their protein profiles: group (G) 1 represented the youngest and the least afflicted patients, and G6 the oldest and critically ill patients. At least two major pathophysiological schemes were indicated based on differing involvement of the kallikrein-kinin system (KKS), the RAS and complement activation. The serum angiotensinogen concentration increased with disease severity. (4) Conclusions: The important role of the RAS in the response to COVID-19 infection was substantiated, but other pathways such as the KKS, plasminogen activation and complement activation influence the systemic response to the infection.

## 1. Introduction

The involvement of the renin–angiotensin system (RAS) in the pathophysiology of coronavirus disease 2019 (COVID-19) is commonly discussed, because angiotensin-converting enzyme 2 (ACE2) is a functional receptor of SARS-CoV-2, and at the same time, a counter-balance for ACE in the RAS [1]. More information on this topic is available in the companion paper [2] and the references therein. We have recently shown that ACE and carboxypeptidase N (CPN) serum activity correlated with disease severity in a cohort of 45 hospitalized and 26 convalescent COVID-19 patients [2]. The degradation of a substrate of these enzymes, the neuropeptide bradykinin (sequence RPPGFSPFR; dabsylated bradykinin—DBK), in serum was used as a readout in those experiments. DBK degradation and CPN activity were significantly reduced in COVID-19 patients and returned to normal during convalescence. The DBK cleavage product generated by ACE, fragment 1–5 (DBK1-5), was significantly increased in critically ill patients and strongly correlated with clinical heart and liver parameters.

In the present study, we used high-definition mass spectrometry (HDMS)-based expression analysis to determine the major proteins and proteases in sera of the same patient cohort in order to shed more light on the ACE and CPN protein network. While blood is a great resource for study, being readily available, it is also an overly complex medium with an extremely large, dynamic range of protein concentration that varies by more than 10 orders of magnitude [3]. Moreover, the most abundant protein species in serum, such as albumin, which itself accounts for ~60% of the total protein, tend to be of the least interest to researchers, which is why proteomic approaches are often based on depleted serum. We used original serum, because we hypothesized that in a systemic disease such as COVID-19, major changes in the protein composition should be detectable at this level. Our goal was to improve the knowledge on the biochemical processes of the infection with respect to the RAS, possibly providing entry points for treatment.

## 2. Results

### 2.1. Patients

This investigation continues a multicenter, prospective observational cross-sectional study of 45 hospitalized patients (HoP) with laboratory-confirmed SARS-CoV-2 infection (nasopharyngeal swab and test by polymerase-chain reaction), admitted to the University Hospital Münster and Marien-Hospital Steinfurt in Germany between March and June 2020. Patient details have been described in our earlier publication [2]. Sera of 40 patients (age range 33–81, mean 56.8; only four females) of this cohort were investigated in the present study. Disease severity was defined as critical (presence of acute respiratory distress syndrome, ARDS; n = 20), severe (requiring oxygen supplementation, n = 11) or moderate (no ARDS, no oxygen supplementation; n = 10) at the time of blood collection (during acute phase of disease, within the first 24 h after admission). ARDS was diagnosed according to the Berlin definition (bilateral opacities on chest radiograph, exclusion of other causes of respiratory failure) [4]. Simplified Acute Physiology Score II (SAPS II [5]) was determined on the day of laboratory measurement and used to characterize the physiological conditions of the patients.

Additionally, 26 individuals who had recovered from laboratory-confirmed SARS-CoV-2 infection participated in the study [2], of whom 23 (age range 55–70, mean 58.9; only two females; CoP) were part of the present work. This group of patients had only moderate symptoms. About half of the probands in this group showed a normal response in the DBK serum degradation experiments, as reported in our earlier paper [2] (CoP-NV, n = 13), although the readout values of some participants were similar to those measured for HoP (CoP-DV, n = 10). The control group (HCtr) consisted of eight healthy volunteers (three females, age range 25–38; five males, age range 31–37).

### 2.2. Serum Protein Expression

**Figure 1:**
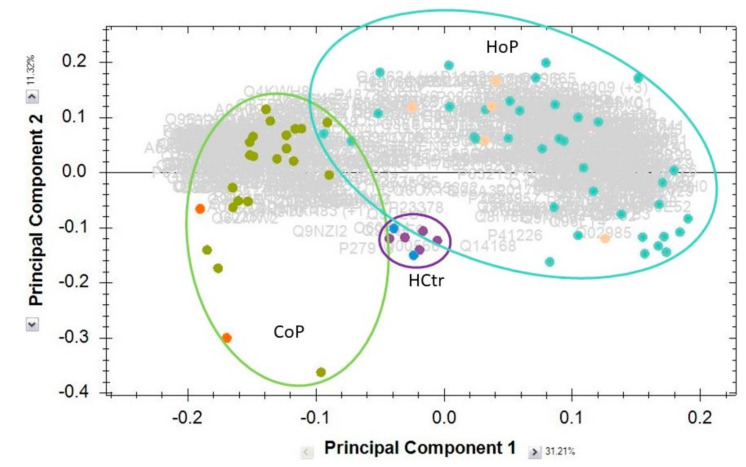
Principal components analysis of shortlisted results from HDMS-based expression analysis (protein assignment by at least two peptides, fold value > 2, ANOVA *p* ≤ 0.05). The majority of the samples came from males. Light (HoP) and dark orange (CoP) and blue (HCtr) dots indicate the samples from the few participating females. Sample groups were separated as indicated by ellipses.

**Figure 2:**
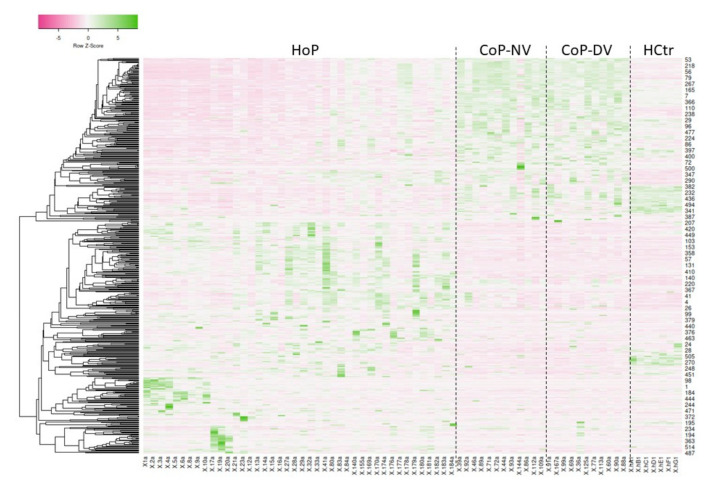
Heatmap of shortlisted results from HDMS-based expression analysis (accumulated ion intensities, protein assignment by at least two peptides, fold value > 2, ANOVA *p* ≤ 0.05; Pearson distance measurement). Shown is sample label vs. protein number.

**Figure 3:**
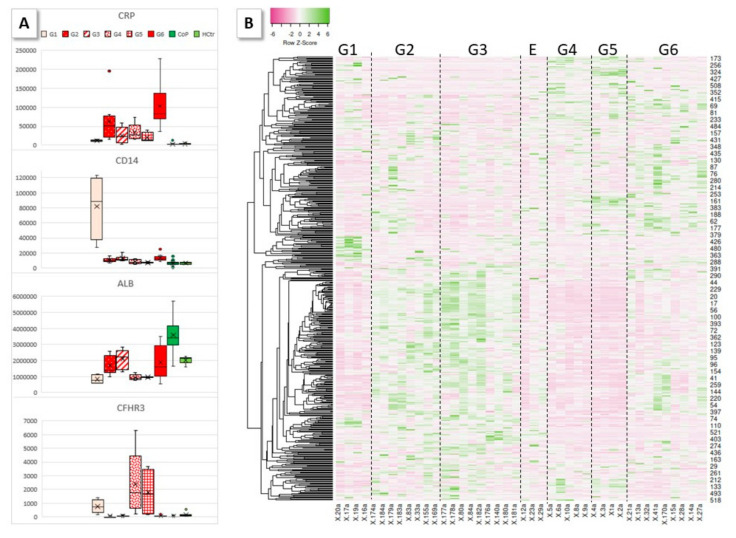
Heatmap of shortlisted results from HDMS-based expression analysis (accumulated ion intensities, protein assignment by at least two peptides, fold value > 2, ANOVA *p* ≤ 0.05; Spearman Rank correlation; sample label vs. protein number) for the HoP group (**B**). Groups G1–G6 were assigned by visual inspection. E represents three samples, which were not considered as a separate group and were excluded from further analysis based on their differing individual protein profiles. (**A**) Boxplots of accumulated ion intensities for CRP (G2, G6), CD14 (G1), albumin (G2, G3, G6, CoP) and CFHR3 (G4, G5) are shown as examples for proteins which were abundant in some groups and not in others.

Sera were tryptically digested and measured by HDMS. In the entire dataset, 523 proteins matched the filter criteria (protein assignment by at least two peptides, fold value of at least two, significance of ANOVA *p* ≤ 0.05) and were analyzed further (for data, see Supplementary Materials, Sheet S1). Principal components analysis (PCA, Figure 1) demonstrated separation of the HoP, CoP and HCtr samples of the hospitalized, convalescent and healthy probands, indicating the presence of differentiating protein factors. The protein list with the up- and downregulated proteins in each case is given in the Supplementary Materials, Sheet S1. In PCA, the samples from females did not separate from those of males, confirming the earlier observation [2] that gender effects were not of importance in these experiments. A glance at the heatmap (Figure 2) shows the reason for the PCA result: the concentrations of a great number of proteins differed considerably among HoP, CoP and HCtr, as shown by the areas with matching colors. Interestingly, HCtr and CoP (NV and DV) presented largely as uniform groups, whereas in HoP, distinct protein and sample subgroups were noted. The protein patterns among the HoP samples differed too much for a reasonable joint analysis against CoP and HCtr (Figure 3B). Therefore, by visual inspection, groups G1–G6 were assigned, each of which contained samples, which roughly shared the same protein profiles. Three samples (E) were excluded, because they did not seem to fit into any group or form their own. Boxplots for selected proteins (CRP-G2, G6; CD14-G1; albumin-G2, G3, G6, CoP; CFHR3-G4, G5) exemplarily demonstrate the major differences among groups (Figure 3A). Complement factor H-related protein 3 (CFHR3), e.g., was one of several proteins that distinguished G4 and G5 from the other groups; it has been associated with the atypical hemolytic-uremic syndrome and defects in the complement alternative pathway [6,7].

### 2.3. HoP Serum Protein Profile Groups

**Figure 4:**
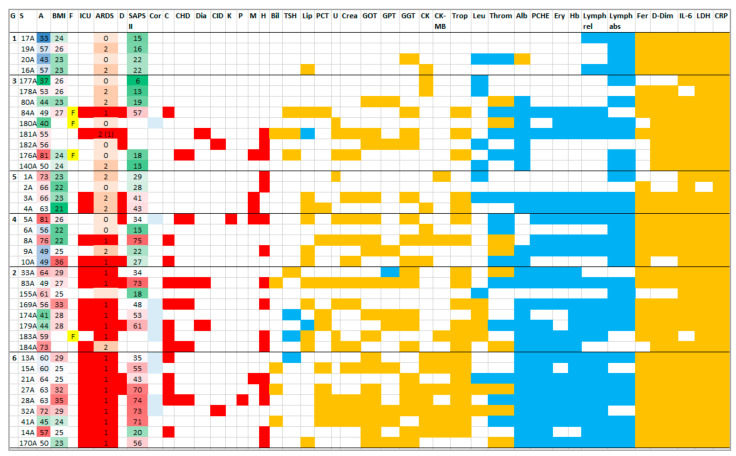
Visual matrix of patient data and clinical parameters (for details, see [2]). G, group; S, sample; A, age; BMI, body mass index; F, female; ICU, intensive care unit; ARDS (1), disease severity (0-no O_2_, 2-with O_2_); D, death; Cor, cortisone treatment; C, coinfection; CHD, coronary heart disease; Dia, diabetes; CID, chronic inflammatory disease; K, kidney disease; P, pulmonary disease; M, malignome; H, arterial hypertonia; Bil, bilirubin; Lip, lipase; U, urea; Crea, creatinine; Trop, troponin; Leu, leucocytes, Throm, thrombocytes; Alb, albumin; Ery, erythrocytes; Lymph, lymphocytes relative/absolute; Fer, ferritin; D-Dim, D-dimers. Orange—values higher than normal, blue—values lower than normal. Red—presence of co-morbidity and a general marker for death, ICU, high BMI and old age. Light blue—cortisone treatment. Green-to-red scale for SAPS II, BMI and age.

**Figure 5:**
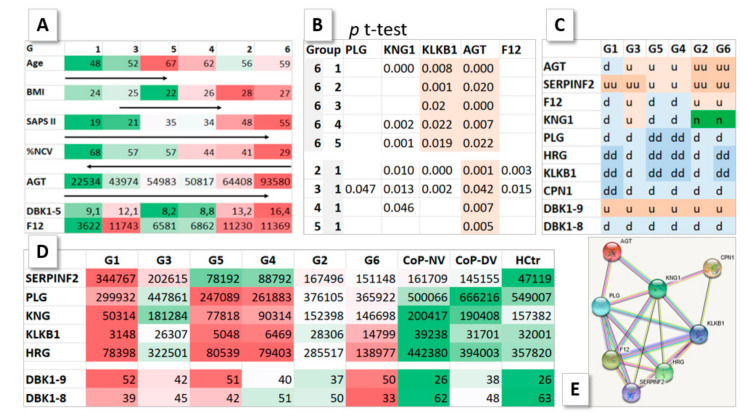
Properties of G1–6. (**A**) Average values for patient parameters (color series: red, worst values; green, best values). In the order G1–G3–G5–G4–G2–G6, the percentages of normal clinical values (%NCV) decreased and SAPS II increased. Average ion intensities for angiotensinogen (AGT) also rose in the series, as did DBK1-5, which additionally showed a second peak in group 3—matching enhanced ion intensities of coagulation factor 12 (F12, Hageman factor) in G3. (**B**) *T*-test results for significant differences in HDMS ion intensities of selected proteins. The AGT concentration differed between G1 and G6 and all other groups. (**C**) Matrix to visualize concentration differences for selected proteins as determined based on their HDMS ion intensities and the DBK degradation results [2] with respect to HCtr. n, normal; d, downregulated (low values); dd, more downregulated; u, upregulated (high values); uu, more upregulated. U for DBK1-9 means low DBK degradation capacity. (**D**) Average HDMS ion intensities and DBK results [2], color-coded (green—normal). (**E**) Network of the eight selected proteins formed by String analysis.

Having noted the differences in the protein patterns in HoP sera, the question arose of whether any correlations with the patient information and the clinical data could be detected which could define each group. The available information (for details see [2]) was assembled in the color matrix shown in Figure 4, highlighting the respective features of groups G1 to G6. While all patients were very ill and thus hospitalized who had increased C-reactive protein (CRP) values, not all required intensive care. In fact, G1 combined those patients who seemed to be the least afflicted by the disease. They had no co-morbidities, did not develop ARDS and were found to have comparatively low SAPS II values (19 on average, Figure 5A). Most of their clinical parameters were normal except for lymphocytes (abs.), ferritin, D-dimers, IL-6, LDH and CRP, along with a few more individually deviating parameters. In contrast, G6 collected patients with critical disease: all had ARDS and required intensive care, and they had, as a group, the highest SAPS II values (55). Almost all clinical parameters were out of range for all members of the group, with few exceptions (e.g., low TSH and high lipase in only one patient each).

The reasons for the uniqueness of G2–G5 were not as clear-cut. G2, for instance, almost paralleled G6 except for a slightly lower average SAPS II (48), normal CK values and better readings in more clinical parameters, such as CK-MB (creatine kinase in heart muscle cells, high in only one patient). Non-normal TSH and lipase values were detected in about half of the group members (3/3, 5/8). G3 seemed to host almost all females and CK-MB was normal. The general condition of the patients was better than in G2, as expressed in the percentages of the normal clinical parameters (%NCV, Figure 4 and Figure 5A). G4, in contrast to G5, had more patients with co-morbidities and considerably less normal clinical parameters. Bilirubin and TSH were normal in both groups; further, leucocytes were normal in G4 and GPT in G5. Detailed inspection revealed an increase in disease severity in the order G1–G3–G5–G4–G2–G6. As is shown in Figure 5A, SAPS II increased in that series while the general condition of the patients worsened as expressed in %NCV. Roughly, in this series, age and BMI increased.

The comparison with our earlier data [2] (Supplementary Materials, Sheet S2_DBK) demonstrated that the formation of ACE-dependent fragment DBK1-5 was highest in G2, G3 and G6. ACE was not detected in the expression experiments using undepleted sera, but the ion intensities of angiotensinogen (AGT), the precursor protein in the RAS, increased from G1 to G6 (Figure 5A). Antihypertensiva-taking patients were distributed across all groups.

### 2.4. DBK Degradation and Protein Expression

**Figure 6:**
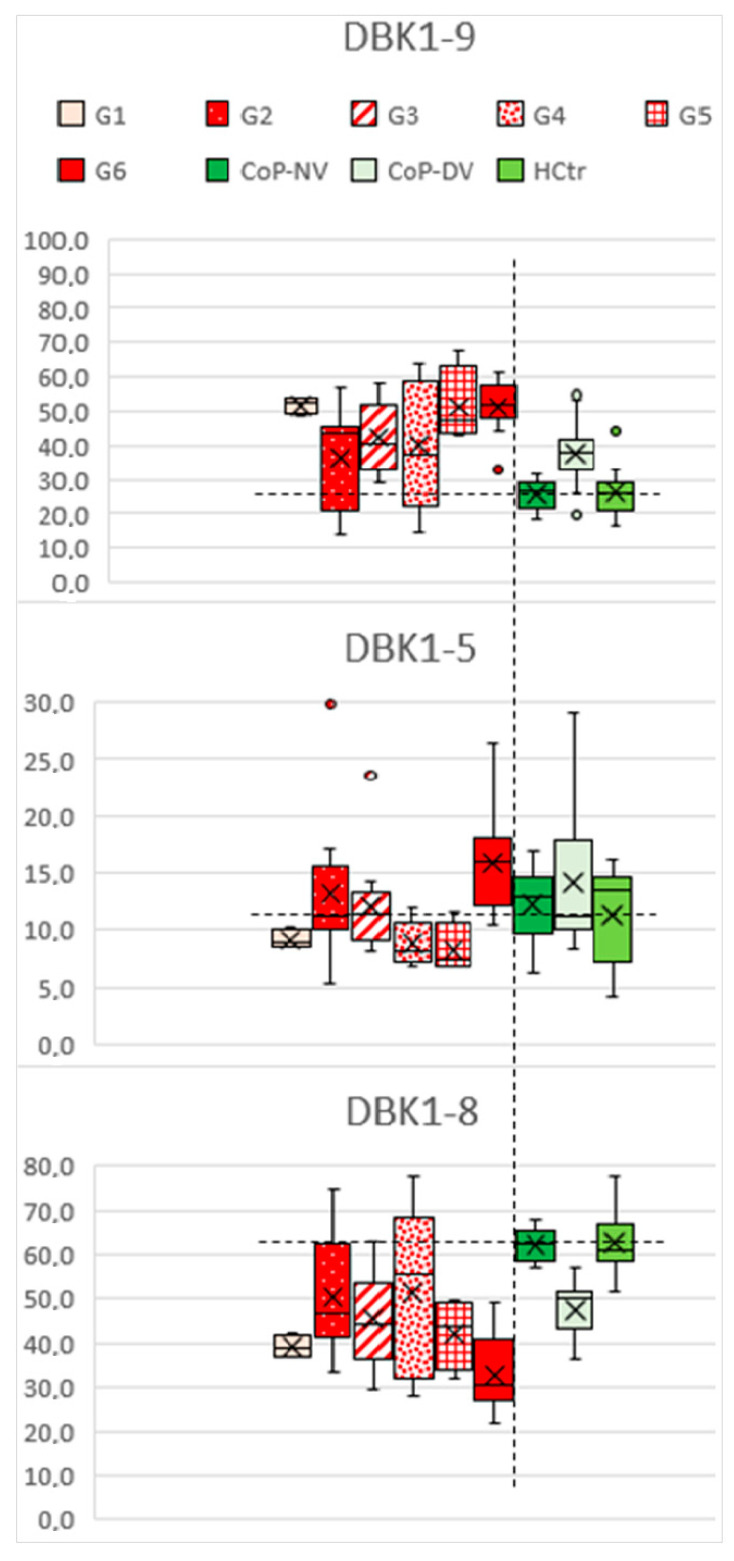
Re-analysis of data for DBK serum degradation (relative spot intensities in thin-layer chromatography) from the earlier publication [2] in groups G1 to G6 for visualization of the impacts of serum proteases such as ACE and CPN. The horizontal dashed lines indicate the mean for HCtr for improved data comparison; the vertical dashed line separates HoP from CoP/HCtr. Sequence of bradykinin: RPPGFSPFR.

**Figure 7:**
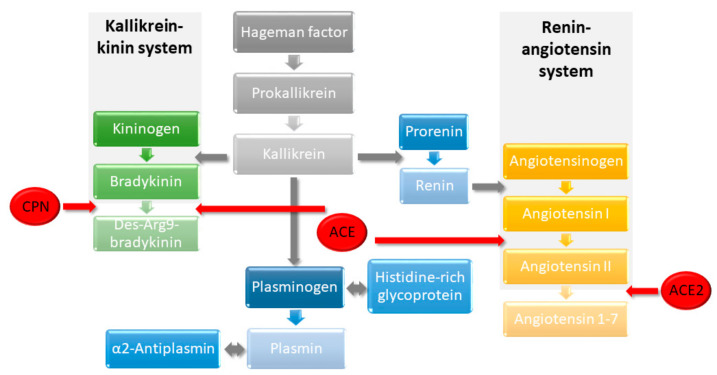
Schematic of the extended RAS and KKS networks, which are connected by ACE. Vasoactive bradykinin is formed from kininogen (KNG1), assisted by kallikrein (KLKB1) and degraded by CPN. KLKB1, which needs Hageman factor (complement factor 12, F12) to be formed from its precursor, also influences the RAS by catalyzing the cleavage of prorenin to renin, which in turn is needed for the formation of angiotensin I from angiotensinogen (AGT). The subsequent formation of angiotensin II by ACE ensures blood pressure homeostasis and is counterbalanced by ACE2, which cleaves angiotensin II. KLKB1 can furthermore activate F12 and plasminogen (PLG). Plasmin degrades fibrin, initiating fibrinolysis, and is inhibited by α2-antiplasmin (SERPINF2). Histidine-rich glycoprotein (HRG) binds PLG and thrombospondin.

**Figure 8:**
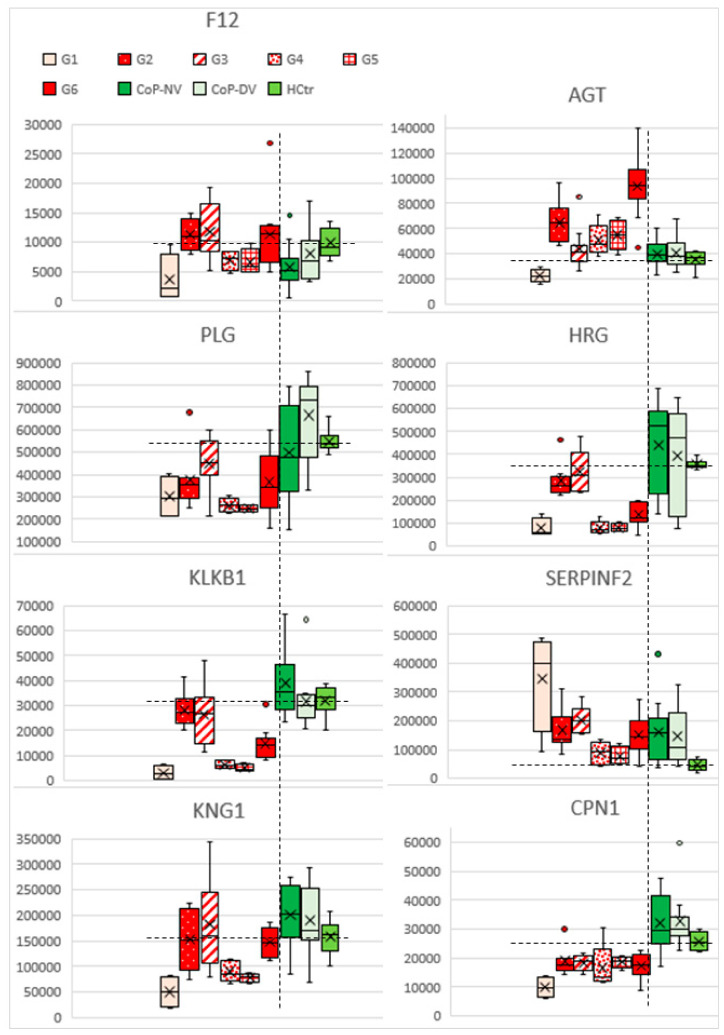
Boxplots of the accumulated ion intensities of eight selected proteins of the RAS and KKS networks (for illustration, see Figure 7 and Figure 5E) for G1–G6. The horizontal dashed lines indicate the mean for HCtr for improved data comparison; the vertical dashed lines separate HoP from CoP/HCtr.

Driven by these new findings, we re-inspected the data from our earlier study [2] with regard to above groups G1 to G6 (Figure 6). Several aspects had to be considered to interpret the results: (1) The serum capacity for the degradation of the neuropeptide reporter DBK was reduced in COVID-19 patients, mainly due to ACE and CPN activity. (2) The formation of DBK1-5 was largely due to ACE and increased with disease severity. (3) The formation of DBK fragments 1–8 mostly resulted from CPN activity and was reduced in HoP. The assay data were compared to HDMS results of selected proteins from the extended RAS network (F12, PLG, KLKB1, KNG1, AGT, HRG, SERPINF2, CPN1—Figure 5; for network schematic, see Figure 7E; for boxplots, see Figure 8).

The most important protein cascades for our experiments are the RAS and the kallikrein–kinin system (KKS), which are connected by ACE (Figure 7). Vasoactive bradykinin is formed in the KKS from kininogen (KNG1), assisted by kallikrein (KLKB1) and degraded by CPN. KLKB1, which needs Hageman factor (complement factor 12, F12) to be formed from its precursor. KLKB1also influences the RAS by catalyzing the cleavage of prorenin to renin, which in turn is needed for the formation of angiotensin I from AGT. The subsequent formation of angiotensin II by ACE ensures blood pressure homeostasis and is counterbalanced by ACE2, which cleaves and thus deactivates angiotensin II. KLKB1 can also activate F12 and plasminogen (PLG). Plasmin degrades fibrin, initiating fibrinolysis, and is inhibited by α2-antiplasmin (SERPINF2). Histidine-rich glycoprotein (HRG) binds PLG, and thrombospondin and may be important for regulating thrombotic influences at vessel surfaces [8]. These eight proteins are by no means sufficient to describe all protein cascades that are involved, but they provide a suitable entry point for further investigations. Their ion intensities in HDMS differed characteristically among groups G1 to G6 (see boxplots in Figure 8, *t*-test results for significant differences in Figure 5B and a visual protein/group matrix in comparison to DBK results in Figure 5C).

DBK serum degradation capacity and DBK1-8 formation were lower than normal in HoP; there were no significant differences among groups (Figure 6). CPN1, the catalytic domain of CPN that is responsible for DBK1-8 formation, correspondingly appeared at lower concentrations in all groups. G1 represented the youngest and the least ill HoP patients. The concentrations of seven of the selected proteins were lower in that group. SERPINF2 was upregulated and the exception. G2 and G6, representing the oldest and most ill patients, showed identical trends for the selected proteins (AGT, SERPINF2, F12—upregulated; KNG1—normal values; PLG, HRG, KLKB1—downregulated). KLKB1 and HRG were significantly less concentrated in G6 than in G2 (Figure 5B). G3 profiles were quite similar except for slightly elevated KNG1, albeit with a large spread. G4 and G5 could not be clearly distinguished based on this analysis, although the DBK degradation capacity and DBK1-8 formation differed slightly (large spread in G4, lower enzyme activity in G5, (Figure 5B and Figure 6).

Incidentally, DBK1-9 and DBK1-8 differed significantly between CoP-NV and CoP-DV [2] (Figure 6). A reason for this observation could not be derived from the expression of above eight proteins, as they did not change much between those two groups. The proteins PLG, HRG, KNG1, SERPINF2, F12 and CPN1 did, however, show much more fluctuation for CoP compared to HCtr, which possibly reflected enzyme re-balancing during the healing process.

### 2.5. Profiles of Regulated Proteins

**Figure 9:**
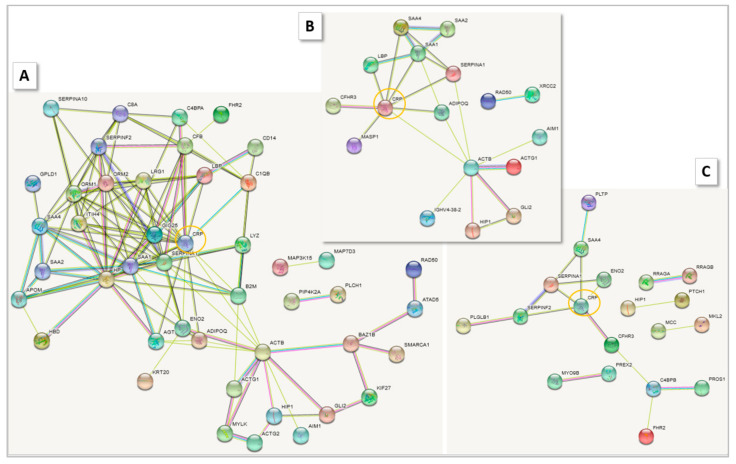
String networks for (**A**) G6, (**B**) G5 and (**C**) G1, of the most abundantly upregulated proteins (confidence at least 50) in the comparison to HCtr. CRP is highlighted.

**Figure 10:**
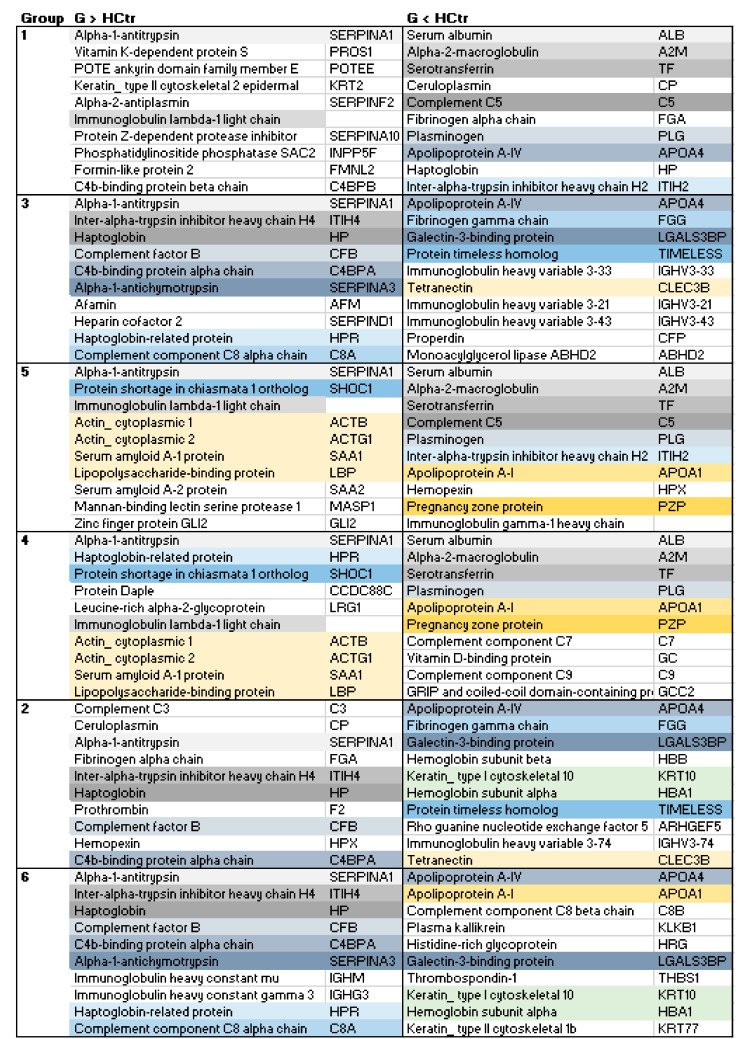
Top 10 most regulated proteins (based on highest confidence) for groups G1 to G6 in comparison to HCtr. Upregulated: G > HCtr, downregulated: G < HCtr. Color-coding highlights the same proteins in different groups.

The profiles of serum proteins determined by HDMS characterized each group in more detail. String analysis of the shortlisted up- and downregulated proteins for the groups G1–G6 vs. HCtr showed that networks of different size, involving CRP, were formed. The smallest were those for the upregulated proteins in G1 (11 members) and G5 (15), and the largest was for G2 (>50) (Figure 9, Supplementary Materials, Sheet S17). For the downregulated proteins, the fewest interactors were detected in G3 (5) and G2 (6), and the most in G1, G4 and G5 (>50). Similarities were evident in the general network structure for G2, G3 and G6 and between G4 and G5, such that the examples in Figure 9 (G1, 5 and 6) can serve as an introductory overview. A number of components were shared among groups, such as the acute-phase protein α-1-antitrypsin (SERPINA1). Figure 10 reports the top 10 proteins for a first glance at the protein composition of the sera (for complete group comparisons with HCtr and CoP, see Supplementary Materials Sheets S3–S15). G1 had the most different protein composition from the other groups featuring, e.g., vitamin K-dependent protein S (PROS1) among the upregulated proteins, a multifunctional anticoagulant [9]. G2, G3 and G6 shared, among other proteins at higher concentrations than in HCtr, acute-phase proteins inter-α-trypsin inhibitor heavy chain (ITIH4), haptoglobin (HP) and complement factor B (CFB), a component of the alternative pathway of complement activation. In G4 and G5, acute-phase proteins serum amyloid A-1 (SAA1) and lipopolysaccharide-binding protein (LBP) were more prominent. Low Hb in COVID-19 patients was associated with critical disease progression before admission; patients with critical illness already had significantly lower Hb levels at the time of hospitalization [10]. Erythrocyte precursors were identified as a direct target of SARS-CoV-2. Dysregulation in hemoglobin and iron metabolism induced by SARS-CoV-2 seemed to be associated with severe COVID-19 courses [11]. In addition, multifactorial anemia can be detected in approximately 95% of generally critically ill patients. Daily sampling to monitor coagulation parameters and acid-base balance may iatrogenically exacerbate anemia [12].

Classification analysis using the PantherDB [13] detected pathways such as angiogenesis, blood coagulation, Wnt signaling and chemokine and cytokine-mediated inflammation as enhanced processes in all groups. Other processes were not common to all groups. Glycolysis and the PLG activating cascade, for instance, were not reported for G5. Overall, the number of affected pathways as an indirect measure for the system disturbance was highest for the proteins upregulated in G2 and G3 (55 each, G1: 25, G4: 31, G5: 39, G6: 36), and for the proteins downregulated in G1 (69), G4 (75) and G6 (64; G2: 30, G3: 22, G5: 56). Interestingly, angiotensin II-stimulated signaling through G proteins and β-arrestin was affected in all groups, but to different degrees: in G1 the detected contributing proteins were downregulated; in G2 to G5, upregulated; and in G6, dysregulated (both up- and downregulated members were detected).

Convalescent patient sera differed from HCtr sera in the 233 proteins reported in Supplementary Materials Sheet S16. More abundant in CoP were the aforementioned HP, PROS1 and SERPINF2, but also afamin (AFM), a pleiotropic glycoprotein involved in various disease states [14], fibulin-1 (FBLN1) and haptoglobulin-related protein (HPR), a high-affinity hemoglobin-binding protein [15]. Downregulated was, among other proteins, properdin (CFP), a plasma glycoprotein that activates the complement system of the innate immune response.

## 3. Discussion and Conclusions

HDMS expression analysis of undepleted patient sera revealed, expectedly, that their protein profiles differed from those of convalescent and healthy probands significantly as a result of the systemic response to the coronavirus (Figure 2). What was unexpected was the presence of six groups into which the HoP samples could be divided according to their protein profiles (Figure 3). These differed to such extent that a joint analysis of HoP vs. CoP and HCtr would not have been reasonable. As it turned out, the distinct protein profiles characterized patients of different disease severity with different pathophysiological responses. Obviously, this additional group separation reduced the power of the analysis, so the results need to be interpreted with care.

All patients had been hospitalized as a result of a strong reaction to the infection, and all (with very few exceptions, Figure 4) shared excessive values for laboratory parameters such as CRP, LDH, ferritin, D-dimers and IL-6. However, within HoP, based on their protein profiles, group G1 represented the youngest and the least ill patient population, whereas G6 collected the critically ill patients, who were, on average, older than 55 and overweight. Many acute-phase proteins were upregulated in G6 sera, including SERPINF2, SERPINA3, SAA1/2/4 and ITIH4, weaving a complicated protein network (Figure 9A). In contrast, in G1, more proteins were found at lower concentrations than in HCtr (Supplementary Materials Sheet S17), and the String network of upregulated proteins was rather rudimentary (Figure 9C). Only in G1 was AGT downregulated. Reductions in AGT and RAS over-activity have been reported to result from physical exercise [16], so one factor for the better disease progression in G1 may be the fitness of the patients.

G1 and G6 framed all other groups, as summarized in Table 1. G2 represented mostly critically ill patients with slightly better SAPS II values and NCV (41%) than G6. Again, G6 represents patients who were, on average, older than 55 and overweight. The general protein profiles of G2 and G6 were similar and shared, e.g., upregulated F12. The equivalent to G2 in terms of the activated protein pathways was G3, which collected younger patients with better SAPS II values. In addition to F12, KNG1 was upregulated there. G4 and G5 differed considerably in their protein profiles from the other groups. The contact activation system did not seem to play the same dominant role as in G2, G3 and G6; and KKS members KNG and KLKB1 were present at reduced concentrations (Figure 5D). The groups differed slightly by the higher average BMI in G4 and the fact that no G5 patients were in critical condition.

It will take further targeted studies, especially of enzymes such as renin, ACE2 and neprylisin, to disentangle the complex pathophysiological mechanisms behind these findings, in particular with respect to co-morbidities, medication and lifestyle habits. What we learned is that the serum AGT concentration correlated with disease severity and that angiotensin II-stimulated signaling seemed to be dysregulated. The considerably lower concentrations of members of the extended RAS and KKS network (Figure 5D) distinguished G1, G4 and G5 from G2, G3 and G6, indicating less involvement in the disease response. Although age and BMI correlated with disease severity, they were not directly associated with the detected changes in protein patterns; neither were co-morbidities, a fact which is best seen in G3, G4 and G5. It appears that at least two major pathophysiological schemes were involved in differing KKS/RAS activity, and possibly, so were defects in the complement alternative pathway, as indicated by findings such as increased CFHR3 in G4/5.

This study suffered from limitations. For one, it was started in the midst of the coronavirus pandemic and worked with the patients as they were admitted to the hospital. As younger people and females were less affected by the disease, the cohorts were not as well matched as we wished. Additionally, we did not have full control on the time of sampling during the course of disease in each patient, because patients appeared at the hospital at varying symptomatic stages. Instead, the investigation offers a glimpse at the current situation in our clinic, and nevertheless provides valuable data on critically ill patients. Furthermore, conclusive assessment of the fluid status of the patients was not possible. It may have an influence on the RAS activity and also change fold values of individual proteins.

The elucidation of the biochemical processes activated in the human body in response to a coronavirus infection will require the study of more clearly defined patient subgroups in order to narrow down the number of affected pathways, but it is a worthwhile endeavor toward the goal of developing personalized treatment options.

## 4. Materials and Methods

### 4.1. Patients and Samples

Serum samples were obtained from each participant after they provided informed consent. The Ethics Committee of Münster University approved the current study (local ethics committee approvals AZ 2020-220-f-S and AZ 2020-210-f-S), and the procedures were in accordance with the Helsinki Declaration of 1975 as revised in 1983. Samples were protected from light and frozen (−80 °C) until measurement.

### 4.2. Protein Isolation and Analysis

Sera (3 µL) were processed as described using filter-aided tryptic digestion [17]. Peptides were dissolved in 30 µL 0.1% formic acid containing 5% acetonitrile, and 0.1 µL was analyzed by reversed-phase liquid chromatography (LC) coupled to high-resolution MS with Synapt G2 Si/M-Class nanoUPLC (Waters Corp., Manchester, UK) using C18 µPAC columns (trapping and 50 cm analytical; PharmaFluidics, Ghent, Belgium) with a 90 min gradient (solvent system 100% water versus 100% acetonitrile, both containing 0.1% formic acid), as detailed elsewhere [18].

### 4.3. Data Analysis/Statistics

Data were analyzed and PCA was performed with Progenesis for Proteomics (QIP, Nonlinear Diagnostics/Waters Corp., Eschborn, Germany) using the human reviewed Uniprot database (UP000005640, Jan. 2021). Shortlists of the protein output were created by demanding protein assignment by at least two peptides, a fold value of at least 2 and a significance of ANOVA *p* ≤ 0.05. Heatmaps were generated using the Heatmapper software tool [19]. Protein network analysis was conducted with String (String Consortium 2021, ELIXIR Core Data Resource) and NetworkAnalyst [20] and geneontology analysis with the Panther classification system [13]. *T*-tests (two-tailed) were performed using SPSS (Version 26IBM Corp., Armonk, NY, USA).

## Figures and Tables

**Table 1:** Comparison of groups G1 to G6. M, moderate disease; S, severe; C, critical; d, downregulated; u, upregulated; Bil, bilirubin; Leu, leucocytes; CK-MB, creatine kinase, heart muscle cells; TSH, thyroid-stimulating hormone; GPT, glutamate–pyruvate transaminase. OK indicates that no patient in this group had abnormal values for the mentioned parameter.

	G1	G3	G5	G4	G2	G6
**Age < 55**	x	x				
**BMI > 25**				x	x	x
**Condition**	M S	M S C	M S	M S C	M S C	C
**SAPS II**	19	21	35	34	48	55
**AGT d**	x					
**F12 u**		x			x	x
**KNG1 u**		x				
**OK**	12 clin. val.	CK-MB	Bil, TSH, GPT	Bil, TSH, Leu	CK	

## Data Availability

All data are provided in the Supplementary Materials or are available on request.

## Supplementary Materials

The following supporting information can be downloaded at: https://www.mdpi.com/article/10.3390/molecules27082495/s1, Supplementary Excel File: Supplement14012022.

## Sample Availability

Samples were limited and were only available for the authors.

## References

[1] Arnold R.G. (2020). COVID-19—Does this disease kill due to imbalance of the renin angiotensin system (RAS) caused by genetic and gender differences in the response to viral ACE2 attack?. Heart Lung Circ..

[2] Tepasse P.-R., Vollenberg R., Steinebrey N., König S. (2022). High angiotensin-converting enzyme and low carboxypeptidase N activity correlate with disease severity in COVID-19 patients. J. Pers. Med..

[3] Anderson N.L., Anderson N.G. (2002). The human plasma proteome: History, character, and diagnostic prospects. Mol. Cell. Proteom..

[4] Ranieri V.M., Rubenfeld G.D., Thompson B.T., Ferguson N.D., Caldwell E., Fan E., Camporota L., Slutsky A.S. (2012). Acute respiratory distress syndrome: The Berlin Definition. JAMA.

[5] Lucena J.F., Alegre F., Martinez-Urbistondo D., Landecho M.F., Huerta A., Garcia-Mouriz A., Garcia N., Quiroga J. (2013). Performance of SAPS II and SAPS 3 in intermediate care. PLoS ONE.

[6] Pouwm R.B., Gómez Delgado I., López Lera A., Rodríguez de Córdoba S., Wouters D., Kuijpers T.W., Sánchez-Corral P. (2018). High complement factor H-related (FHR)-3 levels are associated with the atypical hemolytic-uremic syndrome-risk allele *CFHR3*B*. Front. Immunol..

[7] Lee B., Kwak S., Shin J., Lee S.H., Choi H.J., Kang H.G., Ha I.S., Lee J.S., Dragon-Durey M., Choi Y. (2009). Atypical hemolytic uremic syndrome associated with complement factor H autoantibodies and CFHR1/CFHR3 deficiency. Pediatr. Res..

[8] Silverstein R.L., Leung L.L., Harpel P.C., Nachman R.L. (1985). Platelet thrombospondin forms a trimolecular complex with plasminogen and histidine-rich glycoprotein. J. Clin. Investig..

[9] Dahlbäck B. (2018). Vitamin K-dependent protein S: Beyond the protein C pathway. Semin. Thromb. Hemost..

[10] Algassim A.A., Elghazaly A.A., Alnahdi A.S., Mohammed-Rahim O.M., Alanazi A.G., Aldhuwayhi N.A., Alanazi M.M., Almutairi M.F., Aldeailej I.M., Kamli N.A. (2021). Prognostic significance of hemoglobin level and autoimmune hemolytic anemia in SARS-CoV-2 infection. Ann. Hematol..

[11] Kronstein-Wiedemann R., Stadtmüller M., Traikov S., Georgi M., Teichert M., Yosef H., Wallenborn J., Karl A., Schütze K., Wagner M. SARS-CoV-2 Infects red blood cell progenitors and dysregulates hemoglobin and iron metabolism. Stem Cell Rev. Rep..

[12] Juárez-Vela R., Andrés-Esteban E.M., Gea-Caballero V., Sánchez-González J.L., Marcos-Neira P., Serrano-Lázaro A., Tirado-Anglés G., Ruiz-Rodríguez J.C., Durante Á., Santolalla-Arnedo I. (2022). Related factors of anemia in critically ill patients: A prospective multicenter study. J. Clin. Med..

[13] Mi H., Ebert D., Muruganujan A., Mills C., Albou L.-P., Mushayamaha T., Thomas P.D. (2021). PANTHER version 16: A revised family classification, tree-based classification tool, enhancer regions and extensive API. Nucl. Acids Res..

[14] Dieplinger H., Dieplinger B. (2015). Afamin—A pleiotropic glycoprotein involved in various disease states. Clin. Chim. Acta.

[15] Nielsen M.J., Petersen S.V., Jacobsen C., Oxvig C., Rees D., Møller H.J., Moestrup S.K. (2006). Haptoglobin-related protein is a high-affinity hemoglobin-binding plasma protein. Blood.

[16] Silva S.D., Zampieri T.T., Ruggeri A., Ceroni A., Aragão D.S., Fernandes F.B., Casarini D.E., Michelini L.C. (2015). Downregulation of the vascular renin-angiotensin system by aerobic training—Focus on the balance between vasoconstrictor and vasodilator axes. Circ. J..

[17] König S., Steinebrey N., Herrnberger M., Escolano-Lozano F., Schlereth T., Rebhorn C., Birklein F. (2021). Reduced serum protease activity in Complex Regional Pain Syndrome: The impact of angiotensin-converting enzyme and carboxypeptidases. J. Pharmac. Biomed. Anal..

[18] Distler U., Kuharev J., Navarro P., Tenzer S. (2016). Label-free quantification in ion mobility-enhanced data-independent acquisition proteomics. Nat. Prot..

[19] Babicki S., Arndt D., Marcu A., Liang Y., Grant J.R., Maciejewski A., Wishart D.S. (2016). Heatmapper: Web-enabled heat mapping for all. Nucleic Acids Res..

[20] Zhou G., Soufan O., Ewald J., Hancock R.E.W., Basu N., Xia J. (2019). NetworkAnalyst 3.0: A visual analytics platform for comprehensive gene expression profiling and meta-analysis. Nucleic Acids Res..

